# mean platelet volume as a marker of sepsis in patients admitted to intensive therapy

**DOI:** 10.1186/2197-425X-3-S1-A871

**Published:** 2015-10-01

**Authors:** A Sanchez-Calzada, JL Navarro, L Delgado, O Torres, A Torres, R Gastelum, P Romano, E Monares, C Galindo, G Camarena, J Aguirre, J Franco

**Affiliations:** ABC Medical Center, Intensive Care, Mexico, Mexico; ABC Medical Center, Orthopedics, Mexico, Mexico; Intensive Care, Mexico, Hospital San Angel Inn Universidad, Mexico

## Introduction

The majority of patients admitted to a intensive care unit has signs of systemic inflammatory response (SIRS) with or without sepsis. Since the dieference betwen morbility and mortality of these two populations is very different is a priority to have markers in order to early differentiate between SIRS with sepsis (S+) and SIRS without sepsis (S-).

## Objectives

To investigate the usefulness of the Mean platelet volume (MPV) for differentiating between S- and S+.

## Methods

Admitted patients with SIRS signs admitted to two intensive therapies from January 2012 to December 2014 were prospectively evaluated and classifyied in S- and S + according to the results of blood cultures, imaging studies, procalcitonin> 2 ng / mL and the opinion of two certified intensivists outside the study.

## Results

A total of 202 cases were included. Gender distribution with 51% (103) females. With a mortality of 12.4% (n = 25). With 50% (n = 101) of the population in the S- group and 50% (n = 101) in the S- group. The only statistically significant differences between the two groups was the SOFA score: S- 8 +/- 2; S + 11 +/- 3 (p < 0.05) and mortality, where all the cases were reported in S +. ROC analysis of MVP was conducted with a cutoff value of < 7.7 fl within day 0 AUC 0.974 (CI 0.955-0.994, p < 0.05), at day 1 AUC 0.970 (0961-0997, p < 0.05), at Day 2 AUC 0.987 (0.971-1.00, p < 0.05),and at Day 3 AUC 0.941 (0903-0978, p < 0.05).

## Conclusions

MVP is a useful tool to differentiate between patients with sepsis and those without sepsis. The best way to rule out sepsis is with MVP < 7.7 fl at days 0 and 3.

**Figure 1:**
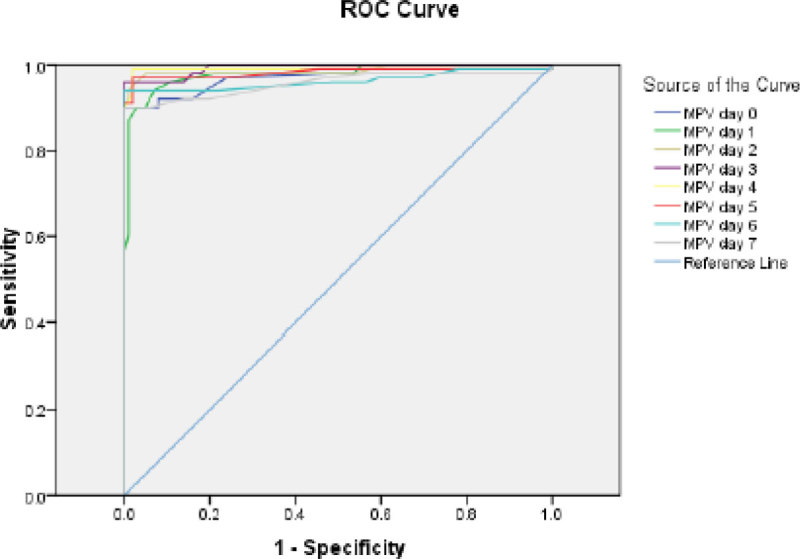
**[ROC]**.

